# Computerized positive mental imagery training versus cognitive control training versus treatment as usual in inpatient mental health settings: study protocol for a randomized controlled feasibility trial

**DOI:** 10.1186/s40814-018-0325-1

**Published:** 2018-08-04

**Authors:** Simon E. Blackwell, Katharina Westermann, Marcella L. Woud, Jan C. Cwik, Torsten Neher, Christian Graz, Peter W. Nyhuis, Jürgen Margraf

**Affiliations:** 1 Mental Health Research and Treatment Center, Faculty of Psychology, Ruhr-Universität Bochum, Massenbergstraße 9-13, 44787 Bochum, Germany; 2 Nexus-Klinik Baden-Baden, Hermann-Sielcken-Straße 80, 76530 Baden-Baden, Germany; 3 St. Marien Hospital Eickel, Marienstraße 2, 44651 Herne, Germany

**Keywords:** Mental imagery, Positive affect, Anhedonia, Cognitive control training, Cognitive bias modification

## Abstract

**Background:**

Enhancing the capacity to experience positive affect could help improve recovery across a range of areas of mental health. Experimental psychopathology research indicates that a computerized cognitive training paradigm involving generation of positive mental imagery can increase state positive affect, and more recent clinical studies have suggested that this training could be used as an adjunct treatment module to target symptoms related to positive affect deficits, specifically anhedonia. The current study investigates the feasibility of adding a positive mental imagery computerized training module to treatment for patients in inpatient mental health settings, with a focus on increasing positive affect and reducing anhedonia. The positive mental imagery training (PMIT) is added to treatment as usual (TAU) in the inpatient setting, and compared to TAU alone, or TAU plus an alternative cognitive training module not hypothesized to increase positive affect, cognitive control training (CCT).

**Methods:**

The study is a feasibility randomized controlled trial with three parallel arms. Up to 90 patients admitted to inpatient mental health treatment clinics in Germany will be randomized to PMIT + TAU, CCT + TAU, or TAU on a 1:1:1 ratio. PMIT or CCT consist of an introductory session followed by up to 8 full training sessions over 2 weeks. All three arms (including TAU) include regular completion of mood measures over the 2-week period. Outcome measures are completed pre and post this 2-week training/monitoring period, and at 2-week follow-up. Data will be presented in the form of both raw means and standardized effect sizes, with 95% confidence intervals, for both intention-to-treat and per-protocol samples.

**Discussion:**

The study will inform feasibility of conducting a fully powered randomized controlled trial investigating the addition of the positive mental imagery training as a treatment adjunct to inpatient treatments for mental health, including potential refinement of study procedures, inclusion/exclusion criteria, and preliminary indications of the likely range of effect sizes.

**Trial registration:**

clinicaltrials.gov, NCT02958228 (date registered: 4 November 2016)

## Background

Treatments in mental health have often tended to focus on the reduction of distress, while neglecting the possibility of enhancing the experience of positive emotions, enjoyment of life, and other aspects of wellbeing [1, 2]. However, there is increasing evidence that such positive facets of mental health may be prognostically important in terms of predicting the course of treatment and recovery across a range of areas of mental health. For example, in the context of depression, lower levels of positive affect have been found to be predictive of poorer prognosis [3], including increased suicidality [4]. Similarly, higher levels of anhedonic symptoms, which may reflect a deficit or dysfunction in positive affect systems [5] have been shown to predict lower rates of remission after pharmacotherapy [6]. Conversely, initial increases in positive affect have been found to predict better rates of remission from pharmacotherapy for depression [7], and experience of positive affect in daily life may buffer against stress [8]. Further, higher scores on a measure of ‘positive mental health’ (“the presence of general emotional, psychological, and social well-being” [9]), which includes several items related to experience of positive affect [9], have been found to predict remission of suicidal ideation in a population sample [10].

Positive affect is also likely to be important for mental health outside of the context of depression; although deficits in positive affect are more associated with depression than anxiety [11, 12], positive affect may in fact also aid recovery from anxiety disorders. For example, in addition to findings relating to suicidality mentioned above, higher levels of positive mental health have also been found to predict better recovery from specific phobias such as animal phobias, fear of heights, and blood-injury-infection phobias [13]. Further, experimental research suggests that higher levels of positive affect may improve extinction learning [14, 15], and thus could be associated with better outcomes from exposure-based psychological therapies for anxiety disorders. None of this data in itself demonstrates directly that increasing how much an individual experiences positive affect, or that targeting the processes underlying positive affect and anhedonia, would improve mental health treatment outcomes. However, it converges on this possibility and indicates that it should be investigated given the potential clinical benefits.

There are many potential ways to try to enhance positive affect over the course of treatment. Although within a depression context there is preliminary evidence that some psychological interventions may increase positive affect [16], in general those aspects of depression related to positive affect such as anhedonia do not respond adequately to current treatments [17, 18]. Thus, one potential route is to develop new psychological treatment packages focused specifically on the putative processes underlying anhedonia and positive affect (e.g., [18, 19]). Another suggestion from recent research is to augment existing treatments via the addition of a brief adjunctive ‘module’ that might specifically target processes relevant to positive affect and anhedonia [20].

The specific treatment adjunct proposed by Blackwell et al. [20] is a computerized cognitive training intervention that involves repeated resolution of ambiguous training stimuli via generation of positive mental imagery. This training paradigm was developed from experimental psychopathology research investigating the effect of mental imagery on emotion [21, 22] and forms part of a broader field of research investigating the experimental modification of cognitive biases as a means to test their potential causal roles in psychopathology, often termed ‘cognitive bias modification’ (CBM, [23, 24]). In the most commonly investigated version of the positive imagery paradigm, training stimuli consist of descriptions of mostly everyday situations, structured so that they are initially ambiguous as to how they might end, but then always resolve positively [25]. An example would be, “As you get out of bed in the morning you think about the day ahead and everything you have to do, and you feel full of *energy and enthusiasm”* (positive resolution in italics). Participants listen to the training scenarios through headphones and are instructed to imagine themselves in the scenarios as they unfold, feeling actively (and emotionally) involved in the situations. Experimental studies with healthy volunteers showed that a single session of the training led to increases in positive affect and a tendency to interpret ambiguous information more positively, compared to a comparison condition in which participants were instructed to think verbally about (rather than imagine) the positive training scenarios [25–28]. Initial clinical studies conducted with adults with current major depression found that a week-long training schedule in which participants completed one session per day from home led to reductions in negative interpretations and symptoms of depression, first in a single case series [29], and then in comparison to sham training [30, 31] or waitlist [32] control conditions.

Following these promising initial results in clinical samples, Blackwell et al. [20] conducted a randomized controlled trial (RCT) in which they extended the training schedule from 1 to 4 weeks and investigated its efficacy in comparison to a ‘sham training’ control condition when delivered via the internet, using a sample of 150 adults with current major depression. Contrary to their expectations, they found no difference between the two conditions in the primary outcome of their trial, symptoms of depression as measured by the Beck Depression Inventory—II (BDI-II, [33]) at post-treatment. However, in post hoc analyses using a ‘per-protocol’ sample (*N* = 115), they found that, compared to the control condition, participants completing the positive imagery training showed greater reductions in anhedonic symptoms of depression (using anhedonia items from the BDI-II) over the course of the 4-week training. A further RCT in which a 1-week training schedule was added to the start of a 10-week course of internet-delivered cognitive behavior therapy (CBT) also found no difference in reduction in symptoms of depression between the positive imagery condition and a sham training control condition in intention to treat analyses [34]. However, there were significant between-group differences in per-protocol analyses, and notably these included a greater reduction in anhedonic symptoms (on the BDI-II) in the positive imagery condition compared to control, albeit as a post hoc analysis. Subsequently, a study conducted with a dysphoric sample [35] similarly found a greater reduction in anhedonic symptoms of depression on the BDI-II, this time in planned analyses, amongst participants completing a week-long positive imagery training schedule, when compared to participants completing a sham training condition and a waitlist. This study also found superiority for the positive imagery condition in terms of reduction in total scores on the BDI-II, but found no differences between the positive imagery and sham training control conditions on measures included to specifically focus on anhedonia or relevant processes, the Snaith-Hamilton Pleasure Scale [36] and the Temporal Experience of Pleasure Scale [37].

To summarize, there is preliminary evidence from both experimental and clinical research to support the idea that the positive mental imagery training could potentially enhance positive affect and reduce anhedonia, and thus may have utility as a positive affect-focused treatment adjunct. However, there are a number of open questions in relation to this eventual aim.

First, although the experimental psychopathology research via which the imagery training was developed (e.g., [25–27]) demonstrated that a single session increased state positive affect in healthy volunteers, subsequent clinical studies have not formally measured positive affect, focusing instead on the reduction of depression symptoms and negative interpretation bias (e.g., [29–31]). Therefore, it is not known whether the training can induce transient or sustained increases in positive affect in clinical samples.

Second, there are limitations in relation to the evidence for reductions in anhedonic symptoms (relative to a matched sham training control) observed so far in depressed and dysphoric samples. In two of the three studies above, anhedonia analyses were post hoc and limited to per-protocol samples. Additionally, the reductions in anhedonia (relative to sham training control) were limited to items on the BDI-II, and would also need to be demonstrated on specific separate anhedonia measures. It is possible that the particular anhedonia-relevant measures used by Pictet et al. [35] are not sufficiently sensitive to show changes over short time periods such as 1 week in the context of the positive imagery training, as the items are not idiosyncratic to participants’ own interests and not closely related to the training content. However, more recently a more idiosyncratic measure that can be individually tailored has been developed (the Dimensional Anhedonia Rating Scale (DARS) [38]), and may be more suitable for detecting training-induced changes in anhedonia over a short time-scale. A further limitation to possible interpretations of the anhedonia reductions observed so far is that in two of the three studies [34, 35], this reduction has been in the context of a reduction relative to control in symptoms of depression as a whole; given that the imagery training may have some efficacy in reducing symptoms of depression [39], it is difficult to claim a specific effect on anhedonia in this context. In order to demonstrate a specific effect on anhedonia, ideally the training would be compared to a condition that resulted in equally large reduction in depression symptoms overall (at least in the short term).

Third, despite the potential transdiagnostic utility of enhancing positive affect, clinical studies have been limited to participants identified on the basis of having current major depression or dysphoria (albeit many with comorbid disorders), rather than a broader range of problems such as anxiety disorders.

Fourth and finally, the studies to date leave open many questions about the potential mechanisms via which engaging in the positive imagery training could result in sustained increases in positive affect or reductions in anhedonia. Potential mechanisms include direct effects of the training on enhancing positive affect (cf. [25]), the potential effects of imagery-based simulation on behavior [40] or reward sensitivity [41], potential links between imagery-based simulation and future anticipation [20], or upregulation of brain areas implicated in positive affect [42]. Understanding the relevant mechanisms could help develop better targeted and more efficacious treatments, and also help inform how best to combine treatment approaches.

The current study is a first step in investigating whether the positive mental imagery training (PMIT) could provide a useful adjunct to treatments across a range of mental health problems by targeting processes thought to underlie positive affect, anhedonia, and positive mental health. Specifically, this study investigates adding the PMIT module to treatments for mental health delivered in an inpatient clinic setting, following studies successfully applying a cognitive training intervention in an inpatient setting in the context of alcohol dependence (e.g., [43, 44]). The study investigates the transdiagnostic applicability of the training by using as a study population patients admitted for treatment of mental health problems to participating inpatient treatment clinics, who may have a mixture of affective disorders, psychosomatic disorders, substance use disorders, and anxiety and emotion dysregulation disorders, without specifying a particular diagnosis as an inclusion criterion. Although we have no a priori reasons to expect that the interventions under investigation would be harmful for particular diagnostic groups, carrying out a first such transdiagnostic study within inpatient settings reduces the risk from potential iatrogenic effects as patients are relatively well-monitored.

The study is a feasibility randomized controlled trial (RCT) run as a small-scale version of a potential future efficacy RCT. Participants are randomized to one of three trial arms. In the treatment arm of primary interest, the clinic’s treatment as usual (TAU) is supplemented by a 2-week positive mental imagery computerized training schedule (PMIT condition). In the second arm, TAU is supplemented by an active comparator 2-week cognitive training schedule (CCT condition), specifically a form of cognitive control training (e.g., [45, 46]). CCT is chosen as a comparator as it would be hypothesized to reduce symptoms of depression, like PMIT, but would not be hypothesized to specifically enhance positive affect or reduce anhedonia. The third arm is TAU (TAU condition), in which participants receive TAU in the clinic, with additional repeated self-report measures of mood, cognitions, and behavior over the 2-week training period (termed “monitoring”). The study aims to establish potential feasibility for this research and to inform future research decisions and design of potential future efficacy RCTs. Such a feasibility study is necessary as the proposed research involves several departures from previous work, such as the transdiagnostic application, the outcomes of interest, the treatment setting, training schedule, and comparison conditions. It also provides opportunity to adapt the study protocols (if necessary) to the new application of the training. The study aims to recruit sufficient participants to allow preliminary estimations of efficacy, within the constraints of the limitations of the trial design.

## Methods/design

### Design

The study is a randomized controlled feasibility trial with three parallel arms using a 1:1:1 allocation ratio. Study arms are TAU + positive mental imagery training (PMIT condition), TAU + cognitive control training (CCT condition), and treatment as usual (TAU condition). Outcomes are measured pre- and post-training, and at a 2-week follow-up. Mechanism measures are repeated throughout the training process. The researcher conducting data collection procedures, including outcome assessments, is not blind to participant allocation.

### Setting and location

The study will take place in clinics offering inpatient treatments for mental health in Germany. The initial study site was the Nexus Klinik in Baden-Baden, Germany, with a first episode of recruitment from November 2016 to January 2017. The St. Marien Hospital Eickel, Germany, was added as an additional site in March 2017, with recruitment starting in April 2017.

The Nexus-Klinik Baden-Baden is a private clinic for psychosomatic medicine, psychotherapy and psychiatry. There are 49 therapy places available for patients suffering mainly from burn-out and stress disorders, depression, anxiety disorders, psychosomatic illnesses, chronic pain, somatoform disorders, stress and adaptation disorders as well as grief reactions, posttraumatic stress disorder, sleeping disorders, obsessive compulsive disorder, comorbid harmful use of psychotropic substances, disorders relating to sexuality, and eating disorders. The average treatment duration in 2016 was 43 days.

The St. Marien Hospital Eickel in Herne is a clinic for psychiatry, psychotherapy, and psychosomatic medicine. There are 159 therapy places available in the inpatient setting for patients suffering mainly from depression, paranoid schizophrenia and alcohol addiction, as well as anxiety disorders, bipolar disorders, traumatic- and stress-related disorders, somatic symptom disorders, schizoaffective disorders, substance-related and addictive disorders, neurocognitive disorders, and personality disorders. Patients are generally referred by their general practitioner or psychiatrist, or otherwise self-present. The average treatment duration in 2016 was 23 days.

### Participants and recruitment

The study will be open to patients admitted to a participating clinic during a recruitment period who meet the study inclusion/exclusion criteria. Inclusion criteria are aged 18 or above, sufficient German language skills, and receiving treatment in the inpatient clinic during the timeframe of the study (i.e., able to complete the training entirely within their admission). The exclusion criterion is existence of a condition or circumstances that would interfere with completion of the study procedures (e.g., severe visual impairment, neurological problem, acute psychosis, or substance withdrawal symptoms).

If a patient is judged not eligible at assessment or admission, they may be re-assessed for eligibility at a later time-point, e.g., if acute symptoms have improved sufficiently to enable participation. Decisions about such potential re-assessments will be made on an ad hoc basis by the researcher carrying out the assessments in consultation with the PI. The inclusion/exclusion criteria are deliberately broad to avoid making potentially premature assumptions about for which patient groups the study may or may not be suitable; inclusion/exclusion criteria (including the potential provision for re-assessment of patients for eligibility, and potential inclusion/exclusion on the basis of specific diagnoses) may be refined and more precisely specified for future studies based on the current feasibility study.

Patients admitted to the clinic will be informed about the study, for example, via announcements at group meetings in the clinic by the study researchers or clinic staff, or individually via study researchers or clinicians providing their care, and those who are potentially interested will be provided with the information sheet and offered a first (eligibility) study assessment appointment. Participants are informed that the study is investigating the potential of two different cognitive training interventions to improve treatment outcomes, but are not informed about the specific rationale for the study in relation to positive affect or potential differential effects of the training programs. Correspondingly, the official title of the study, as used on study materials (“The effect of computerized cognitive training on mood and thinking style amongst patients in inpatient settings”), does not mention positive affect, mental imagery, or anhedonia.

### Sample size

The primary aim is establishing feasibility of conducting the research and delivering the intervention, and as such the sample size is determined by issues of pragmatics rather than statistical power [47, 48]. For this study, a period of testing from the study start until approximately March 2017 was initially planned, but this was then extended up to potentially the end of March 2018 to allow recruitment of a larger sample. All patients admitted during the recruitment period, i.e., while research staff are present and have availability for appointments, will be informed about the study and offered the opportunity to take part. It is anticipated that sufficient number of participants will be recruited (approximately 90) during this time to establish feasibility and provide initial estimates of effect sizes (i.e., 30 per group, following the suggestion of having at least 30 participants to provide an estimate of a parameter [48, 49]). However, such a sample size would not be expected to be sufficient to detect between-group differences. If the research appears to be feasible, the sample size for subsequent RCTs would be informed by formal power calculations.

### Interventions

#### Positive mental imagery training (PMIT)

The positive mental imagery training (PMIT) is a cognitive bias modification (CBM) paradigm adapted from that developed via experimental psychopathology work (e.g., [25]) and previous clinical studies in the context of depression (e.g. [20, 29]). Participants listen to training stimuli, which consist of descriptions of (mostly) everyday scenarios, and imagine themselves in each scenario as it unfolds. The descriptions are structured so that they start ambiguous (i.e., could end positively or negatively), but always resolve positively. The aim is that via repeated practice in imagining positive resolutions for ambiguous situations, a more adaptive bias is trained to automatically imagine positive resolutions for ambiguous situations in daily life. Further, imagining the positive resolutions leads to generation of congruent positive emotions, and thus engaging in the training may induce increases in state positive affect (e.g., [25]).

The training sessions consist of 5 blocks of 8 training scenarios, which results in each session taking approximately 15 min. The initial introductory session includes an extended introduction to mental imagery with several guided imagery examples (on the computer) followed by 4 blocks of 5 scenarios. The introduction is computer-administered to reduce the extent to which the intervention requires input from a trained researcher/clinician and enhance the ease of potential future replication and dissemination.

Following suggestions from previous research [20, 35], to increase engagement in generating vivid imagery over the course of a multiple-session intervention, participants receive graphical feedback of their ‘performance’ (mean, maximum, and minimum vividness ratings) after each block of scenarios. Participants are prompted to reflect on their ratings and use them to try to improve or maintain the vividness of their imagery over the subsequent block.

The program is implemented as a desktop application written in Java. The training scenarios were newly developed for the current study along the lines of those used in previous research, but with more focus on specifying specific emotions associated with the positive resolution (to enhance generation of positive emotions via imagery), and to be more generic in their settings/locations to enhance applicability across different samples and patient groups.

#### Cognitive control training (CCT)

The cognitive control training (CCT) is an adaptive Paced Auditory Serial Addition Task (PASAT), adapted from that implemented in several studies investigating cognitive control training in the context of depression/rumination (e.g., [45, 46, 50]).

Participants are presented via headphones with a continuous stream of digits (1–9), with the task of immediately calculating the sum of the last two digits heard and clicking a corresponding button (1–18). Each session starts with an inter-stimulus interval (ISI) of 3000 ms, which is reduced by 100 ms following four consecutive correct responses or increased by 100 ms following four consecutive incorrect responses. Thus, the difficulty of the task is adapted to the participant’s performance and increases as their performance improves. Participants receive feedback on their performance via presentation of their ISI and number of consecutive correct/incorrect responses throughout the task. Each session consists of 400 trials (to result in a session length approximately equal to that for the PMIT). Participants can ‘pause’ the task at any time to take a break if desired. Training success is indicated by the decrease in ISI over the course of training. The CCT program is implemented as a desktop application written in Java.

Preliminary research has indicated potential for the adaptive PASAT in reducing symptoms of depression amongst people with current or remitted major depression in conjunction with an attention training paradigm [46, 50, 51], and on its own [45, 52, 53].

#### Treatment as usual (TAU)

Treatment as usual (TAU) may include individual and group psychotherapy sessions and other therapeutic activities in the clinic, and possible pharmacotherapy. The treatment offered in the participating clinics follows primarily CBT principals and includes a multimodal range of intensive individual and group therapies. This can be complemented by sports, movement, relaxing, music, ergo, creative, and art therapy, according to the individual patients’ needs. Participants in all conditions receive TAU.

Participants in the TAU condition also complete “monitoring,” a set of computerized self-report questions about mood/cognitions over the previous day (see the Measures section below) on the computer 8 times over the 2-week intervention period (< 5 mins per completion). These questions are also included at the start of every training session in the other two conditions. There are two main reasons for including these monitoring questions in the TAU group. First, monitoring can in itself have beneficial effects, e.g., via repeated reflection on one’s own progress during treatment, and completing the computerized questionnaires involves a meeting with the research staff. Including the “monitoring” in the TAU group therefore allows it to also control for these non-specific aspects of the intervention groups. Second, completion of the monitoring in the TAU group provides data on the trajectory of these measurements over time in the absence of the cognitive training interventions, providing a comparison for the other conditions. The program for the monitoring ratings is implemented as a desktop application written in Java.

### Measures

See Table 1 for a tabular display of the measures and their schedule of administration. Additionally, information on patient enrolment, adherence, and attrition rates will be collected to inform decisions around feasibility and presented as part of publication of the trial results, although no formal specific feasibility outcome was pre-specified.

**Table 1:** Study measurement schedule

	Eligibility	Pre-training/baseline	During training	Post-training	Follow-up
Consent	x				
Demographics, clinical information	x				
DIPS-/Mini-DIPS-OA	x				
DARS	x			x	x
PANAS-P		x		x	x
QIDS-SR		x		x	x
GAD-7		x		x	x
PMH		x		x	x
PIT		x		x	
SST		x		x	
STIAT		x		x	
CEQ		x			
Feedback					x
Mood monitoring			x		

*DIPS-/Mini-DIPS-OA* Diagnostisches Interview für psychische Störungen-Open Access, *DARS* Dimensional Anhedonia Rating Scale, *PANAS-P* Positive and Negative Affect Schedule–21-item positive subscale, *QIDS-SR* Quick Inventory of Depressive Symptomatology–Self-Report, *GAD-7* Generalized Anxiety Disorder Scale, *PMH* Positive Mental Health Scale, *PIT* Prospective Imagery Test, *SST* Scrambled Sentences Test, *STIAT* Single-Target Implicit Association Test, *CEQ* Credibility/Expectancy Questionnaire

### Primary outcome

#### Positive subscale (21 items) from the extended Positive and Negative Affect Schedule (PANAS-S [54])

This measure of positive affect comprises the joviality, self-assurance, attentiveness, and serenity subscales of the extended PANAS [54], and its use in this study follows previous experimental research examining the effect of positive mental imagery on state mood (e.g., [25–27]). In the current study, the PANAS-P is administered with the ‘past week’ instructions (i.e., participants are asked to rate how much each item applies to how they have been feeling ‘in the past week, that is, during the past 7 days, including today’), and the German translation of the scale [55] is used. The individual positive subscales have shown good internal consistency (Cronbach’s alpha) in a German community sample (joviality: α = 0.92; self-assurance: α = 0.78; attentiveness: α = 0.79; serenity: α = 0.81 [55]). The primary outcome relates to the PANAS-P at post-training (i.e., about 2 weeks post-baseline). The PANAS-P is also completed at pre-training and follow-up.

### Secondary outcomes

#### Dimensional Anhedonia Rating Scale (DARS [38])

The DARS is a self-report measure of different facets of anhedonia (desire, motivation, effort, and consummatory pleasure) across different hedonic domains (hobbies/past-times, food/drinks, social activities, and sensory experiences). In the current study, an extended 26-item version (which includes extra items to measure motivation) provided by the scale authors is used, in a German translation by A. Bieda (Ruhr-Universität Bochum, Germany), in conjunction with SEB and the scale authors. The original 17-item English-language version of the DARS has shown good convergent and divergent validity, and high internal consistency (Cronbach’s alpha) across both community (α = 0.92) and patient (α = 0.96) samples. The baseline administration of the DARS is at the eligibility assessment rather than the pre-treatment assessment for practical reasons (in order to reduce the time spent completing questionnaires prior to completing the introductory training session), and the DARS is repeated at post-training and follow-up.

#### Quick Inventory of Depressive Symptomatology (QIDS-SR [56])

The QIDS-SR was designed as brief self-report measure of depressive symptoms that specifically assesses core criterion DSM-IV symptoms of depression. It has been validated against established clinician rating scales for use in clinical trials [56]. The German translation provided by the scale publishers (http://www.ids-qids.org/) is used, which has shown acceptable internal consistency (Cronbach’s alpha = 0.77) in a sample of depressed German adults waiting for psychotherapy [57]. The QIDS-SR is completed at pre-training, post-training, and follow-up.

#### Generalized Anxiety Disorder Scale (GAD-7 [58])

This measure is a brief (seven items) self-report anxiety questionnaire designed as a screener for Generalized Anxiety Disorder in primary care. The German translation [59], which has shown high internal consistency (Cronbach’s alpha = 0.89) and construct validity in a large general community sample [59], is used. The GAD-7 is completed at pre-training, post-training, and follow-up.

#### Positive Mental Health Scale (PMH [9])

This questionnaire consists of 9 items designed to assess positive mental health. The measure (original in German) has been used in previous longitudinal research and shown to predict remission from specific phobia and suicidal ideation [10, 13]. Psychometric evaluation of the German PMH has shown good psychometric properties in student, patient, and general community samples, including good internal consistency (Cronbach’s alpha across all groups, α = 0.93), convergent and divergent validity, and sensitivity to therapeutic change [9]. The PMH is completed at pre-training, post-training, and follow-up.

#### Prospective Imagery Test (PIT [60, 61])

The PIT is a measure of the vividness with which people can imagine positive or negative events happening in their future. Participants read a list of hypothetical positive and negative future scenarios and are asked to form a mental image of each, before rating how vivid their image is from 1 (not at all vivid) to 5 (very vivid). Each assessment includes five positive and five negative hypothetical future situations, with a different set at baseline and post-treatment. A German translation [62] is used. Internal consistency (Cronbach’s alpha) has previously been good in a mixed (patient and healthy control) German sample for both the positive (α = 0.89) and negative (α = 0.83) subscales [62]. As a mechanism measure, the PIT is completed at pre-training and post-training only.

#### Scrambled Sentences Test (SST [63, 64])

This is a measure of interpretation bias, in which the participants are presented with a set of 20 scrambled sentences (e.g., “winner born I am loser a”) and instructed to write a number above five of the six words in order to construct a grammatically correct sentence, while holding in mind a six digit number (‘cognitive load’). This measures the tendency of participants to interpret ambiguous information either positively (“I am a born winner”) or negatively (“I am a born loser”). Participants will complete different versions at baseline and post-intervention. A German version translated by MLW and KW, with reference to a previous German version [65], will be used. As a mechanism measure, the SST is completed at pre-training and post-training only.

#### Single-Target Implicit Association Test (STIAT [66])

A Single-Target Implicit Association Test will be used to assess automatic associations between positive/negative affect relevant attributes and targets related to the self. Scoring is according to the algorithm by Greenwald, Nosek, and Banaji [67]. As a mechanism measure, the STIAT is completed at pre-training and post-training only.

### Other measures

#### Demographics and clinical information

Participants will complete demographic information, plus information about German language proficiency, whether they are taking any medication (and provide details if so), the duration of their stay in the clinic so far, and the reason they are in the clinic (in a few words).

#### Diagnostisches Interview für psychische Störungen–Open Access (DIPS-OA [68])/Diagnostisches Kurzinterview bei psychischen Störungen–Open Access (Mini-DIPS-OA [69])

The DIPS-OA or the shorted version Mini-DIPS-OA is a structured clinical interview, previous published versions of which have been demonstrated to be valid diagnostic tools for use with both outpatients and inpatients in psychiatric settings (e.g., [70]). The most recent version (for DSM-5) will be used, with a shorted version (Mini-DIPS) used if necessary to shorten assessment times. The structured clinical interview will be audio-recorded, provided that participants provide specific consent for this.

#### Credibility/Expectancy Questionnaire (CEQ [71])

The CEQ is a widely used measure of treatment credibility and expectancy of symptom improvement. A German version adapted from that by Riecke, Holzapfel, Rief, and Glombiewski [72] will be used.

#### Self-report mood/cognitions over the previous day

In order to provide repeated measures of potential mechanisms of change, at the beginning of each training session, participants will make a series of ratings about their mood and cognition over the previous day. Participants will rate the mood words: happy, sad, calm, anxious, optimistic, hopeless, and the following statements “Positive thoughts seem to pop into my head,” “Negative thoughts seem to pop into my head,” “I have been able to look forward to activities and events,” “I have been able to enjoy taking part in activities or events,” “I was an active person and accomplished the goals I set out to do.” (this last item taken from the Behavioral Activation for Depression Scale [73], German version by [74]). Ratings are made on the scale used for the PANAS (i.e., from 1 = not at all to 5 = extremely). These ratings are integrated into the beginning of the training sessions for the PMIT and CCT conditions, and in stand-alone Java program for the TAU condition.

#### State mood change

At the beginning and end of every training session (integrated into the computer program), participants in the PMIT and CCT groups will rate the following words according to how they are feeling “right now/ in the past few minutes”: happy, sad, calm, anxious, optimistic, and hopeless. Ratings are made on the scale used for the PANAS (i.e., from 1 = not at all to 5 = extremely).

#### Feedback questionnaire

At follow-up, participants will complete a feedback questionnaire developed for the study, which includes questions about acceptability and perceived usefulness of the intervention and questions to assess potential demand.

### Procedures

Table 1 illustrates the schedule of measurement and testing. All study assessments are carried out by a member of the research team.

#### Eligibility assessment

Following written informed consent, participants will complete background/demographic information and a structured clinical interview (DIPS-/Mini-DIPS-5), and eligibility criteria will be confirmed. The pre-training/baseline administration of the Dimensional Anhedonia Rating Scale (DARS) will be completed at this assessment (for practical reasons, to shorten the pre-training/baseline assessment).

#### Pre-training/baseline assessment

Participants will complete baseline measures (PANAS-P, PMH, QIDS-SR, GAD-7, PIT, SST, STIAT) and be randomized to one of the three conditions (TAU, PMIT, CCT). Following a brief introduction to their allocated condition, participants will complete the Credibility/Expectancy Questionnaire (CEQ). They will then complete an initial version of their allocated task, which comprises instructions (on the computer) and a brief version of the training.

#### Randomization

Randomization will be stratified by gender and by clinic, using variable block lengths and implementation via sealed envelopes so that allocation will remain unpredictable to trial staff. The randomization sequence was generated via the trial statistician (TN) using the statistical software package R version 3.3.1. Only the statistician will have access to this sequence. The statistician will sign and place the allocations into sequentially numbered opaque envelopes, with the allocation printed in pale ink so that it is not readable through the envelope. The envelope will be signed across the seal and tape placed over the signature.

Randomization will occur in the pre-training/baseline assessment after a participant has completed all baseline measures. The administering researcher will first photograph the envelope seal, then open the envelope, and write the date, time, and participant number on the allocation sheet, then sign and photograph it. These time-stamped photographs thus provide evidence of appropriate allocation and can be cross-checked against the time-stamp for the questionnaire and training data. At the end of the study, the allocations will be checked against the original allocation sequence held by the statistician (TN).

#### Training sessions

Patients in one of the two training allocations will complete eight sessions of the training over a 2-week period, with four sessions planned per week. The exact schedule will be decided (to fit in with the patient’s schedule) with the researcher at the baseline session. Each session is approximately 15 min long. Each session will start with a brief series of questions about mood and cognition over the previous day (see the section *Self-report mood/cognitions over the previous day* above), and state mood will be assessed immediately pre and post each session (see the section *State mood change* above). Participants in the TAU condition will complete only the computerized assessment measures on 8 occasions, with a schedule similar to that for the training sessions (less than 5 min each time).

#### Post-training assessment

After completing the final training session, participants will complete the post-training assessment (PANAS-P, PMH, DARS, QIDS-SR, GAD-7, PIT, SST, STIAT).

#### Follow-up assessment

2 weeks after the post-training assessment, the participant will complete the follow-up assessment (PANAS-P, PMH, DARS, QIDS-SR, GAD-7, Feedback questionnaire). They will read debriefing information and have the opportunity to ask further questions. If the participant has already been discharged from the clinic by this point, they can complete the questionnaires online from home or on paper and post them back to the clinic, and receive the debriefing information via email with the option of telephone contact if they have any final questions about the study.

### Statistical analyses

In line with recommendations for pilot/feasibility studies (e.g., [47]), the purpose of this study is not hypothesis-testing, and thus it is not planned to test the statistical significance of potential differences between treatment arms on the clinical outcome measures.

#### Outcome measures

Outcome data will be presented in the form of both raw means and standardized effect sizes at each time point, with 95% confidence intervals, for both intention-to-treat and per-protocol samples. For scales with subscales (e.g., PANAS-P, DARS), both the total scale score and individual subscale scores will be calculated. Outcome data will be presented both pooled across sites and separately per site.

The intention to treat sample will include all participants randomized to condition, and estimated means, effect sizes (between and within-group, expressed as Cohen’s *d*), and 95% confidence intervals will be estimated via fitting of a linear mixed model over the three assessment time-points (i.e., a mixed model repeated measures ANOVA). Mixed models are used for the intention to treat estimates as this allows inclusion of all participants without the need to impute missing data [75].

The per-protocol sample is defined as those participants who complete at least four of the eight training sessions (or measurement sessions in the TAU condition), including at least one in the second week of training, and who provide the required outcome data. Means, effect sizes, and 95% confidence intervals will therefore be computed from the available data.

#### Process measures

It is planned to examine the data collected within each session (i.e., ratings of mood/cognition/behavior over previous day, change in state mood over individual sessions, performance ratings such as vividness ratings/ISIs) with the aim of devising appropriate statistical analyses for investigating the mechanisms and time-course of change over training to be planned a priori for future studies.

#### Other measures

It is planned to present other measures (e.g., expectancy, feedback) in the form of means with 95% confidence intervals per condition.

### Risks and burdens

There are no foreseen specific major risks associated with the study-specific procedures, all of which have been implemented in clinical populations, e.g., participants with current major depression. While completing the training requires sustained concentration and may be perceived as potentially burdensome by patients, feedback for the PMIT in previous studies has indicated that it is acceptable, and in fact has been generally positive. The sessions are tailored to the potentially reduced ability to concentrate of patients by keeping them short, with frequent/self-paced breaks. To reduce participant burden, relatively brief measures have been chosen for outcome measurement.

The nature of the population (i.e., people requiring treatment for, e.g., emotional disorders) means that suicidal ideation and self-harm are potential (albeit low likelihood) risks. These risks are minimized by the studies taking place within the context of inpatient treatment settings, in which patients receive extensive support and therapeutic input. Study personnel do not have direct responsibility for monitoring and managing such risk, with the exception that if information is provided in the course of completing study procedures (e.g., interviews or questionnaires) that indicates potential risk, the research staff would communicate this to the clinical team responsible for the participant’s care.

### Monitoring

#### Data monitoring

The early-phase exploratory nature of the trial, short time-scale for data collection, and settings within which it takes place means that a formal data monitoring committee (DMC) is not needed.

To realize the aim of the study in establishing feasibility via adapting study procedures as necessary, data related to adherence, feedback, and within-session ratings may be examined as they become available (at least in the initial stage of the study) to allow monitoring of the implementation of study procedures, training engagement etc., and making of adjustments if necessary. The extent or nature of any such adjustments would be reported in the resulting publication of study outcomes. If potential concerns arise, these will be discussed with relevant members of the research team and clinicians at the relevant site if appropriate.

#### Adverse events

The following potential adverse events will be monitored and their occurrence/non-occurrence recorded on an ‘Adverse Event Record’ form completed for each patient: suicidal ideation (≥ 2 on item 12 of the QIDS); worsening of depression/anxiety symptoms (increase of > reliable change index on the QIDS or GAD-7 from baseline to follow-up); dropping out of the inpatient treatment against medical advice; terminating the study due to feeling that it is having adverse effects on mental health; self-reported adverse effects of the study; other events not listed here judged to be adverse events. Adverse events will be communicated to the individuals responsible for oversight of the management of the research at the individual site. Any serious adverse events will be notified to the ethical committee.

If issues of risk are identified during the study (e.g., emergence of suicidal ideation), these will be communicated to the responsible clinicians at the clinic, who will have responsibility for assessing and managing potential risk.

### Ethical considerations

All participants provide written informed consent, and if they decide not to take part in the study or to withdraw, this will not have an impact on the treatment they receive during their admission to the clinic. All participants receive either their treatment as usual or an intervention indicated by previous research to be potentially beneficial (i.e., there are no sham or placebo conditions), and the study design aims to keep measurement burden relatively low. Participants provide consent for anonymous research data (i.e., individual-level participant data) to be made available via publication. There are no financial or other material incentives for participants to take part in the research.

Ethical approval has been provided by the ethics committee of the Faculty of Psychology, Ruhr-Universität Bochum (Germany), approval no. 325.

### Confidentiality and data management

Participants will be assigned a numerical code, and all data collected will be identified by this code (i.e., in anonymized form). A code list containing participant names and linking them to participant IDs will be kept on paper in a secure (locked) location accessible only to study personnel. This code list will be destroyed after the end of the study. Questionnaire data will be collected via the UniPark survey software or on paper, with initial data entry on study laptops. Data will be regularly backed up remotely to servers at Ruhr-Universität Bochum (Germany). Study researchers at Ruhr-Universität Bochum will have primary access to the final study dataset.

Prior to data analysis, 100% of the primary outcome and a random sample of 15% of the secondary outcome data will be checked by a researcher blind to participant allocation. If an error rate > 1% is detected, 100% of the outcome data will be re-scored and re-entered. Paper data will be scanned to PDF for archiving purposes, and the original paper forms will be destroyed as soon as is permitted.

## Discussion

The current study aims to establish potential feasibility of testing a computerized positive mental imagery training (PMIT) schedule as an adjunct to treatment as usual for people receiving treatments in mental health inpatient settings. The study is motivated by the potential value of targeting positive affect and anhedonia, the preliminary indications that the positive mental imagery training may provide a route for doing so, and the limitations of current treatment approaches in this regard. The study results will inform feasibility and design of a potential future efficacy RCT, as well as provide indications as to whether such an RCT would be warranted. Although the study specifically examines the addition of PMIT to inpatient treatment, the results should have broader relevance for investigating potential adjunctive clinical applications of the training.

Although the current study is designed as a small-scale version of a potential future efficacy RCT, there are a number of limitations to the current study that would be addressed in a future study. Due to practical and resource constraints, at any one time at a particular research site all study procedures are carried out by one researcher. This means that the researcher administering the training sessions and outcome assessments is not blind to participant allocation, providing a potential source of bias. For a subsequent fully powered RCT, funding would be sought for personnel to allow blind assessment of outcome measures. It would also be useful for a future RCT to include a longer follow-up period, including diagnostic assessments at follow-up. Additional finer-grained mechanisms measured, for example, momentary assessment of affect [76] or positive imagery in everyday life [77], would also be helpful to build a more comprehensive picture of potential mechanisms. In the current feasibility study, details of the treatment-as-usual received by each individual participant during their participation in the study (e.g., number and nature of psychotherapy sessions received, if any) is not collected, but such information would be useful to collect in future trials where possible. The study sites may not be representative of inpatient clinics in other locations, for example, in relation to the characteristics of the patients admitted and the therapeutic programs, and thus it cannot be assumed that feasibility, efficacy, or safety outcomes will generalize to other inpatient clinics. Finally, we did not pre-specify a formal feasibility outcome (e.g., in terms of rates of recruitment, adherence, or attrition).

A noteworthy aspect of the design of the current study is the choice of comparison conditions for the PMIT. While studies in the broader area of cognitive bias modification often use ‘sham training’ control conditions [78], the current research concerns questions of whether adding PMIT to TAU improves treatment outcomes (thus requiring TAU as a comparison condition), and further testing potential specificity of the effect of PMIT on positive affect and anhedonia. Thus the best comparison condition, in addition to TAU, was thought to be an active comparator (i.e., another active training program) thought to reduce symptoms of depression, but not expected to specifically increase positive affect or reduce anhedonia (apart from potential changes secondary to improvements in depression/anxiety). In an adequately powered RCT, this would allow investigation of specificity of effect of the PMIT on positive affect/anhedonia. A cognitive control training procedure, specifically the adaptive PASAT, was chosen as it appeared to be the cognitive training procedure with the strongest empirical support in the context of depression at the time of the study design.

There are also other potential advantages to using an active comparator rather than a sham training condition in this research context [78]. It removes potential problems of participants trying to guess whether they are receiving a ‘real’ or ‘sham’ training, particularly in an inpatient setting in which participants may ‘compare and contrast’ their respective training conditions. There may be ethical advantages in using an active comparator, in that no patient is required to carry out a “placebo” procedure. This may also facilitate participant engagement in the study (i.e., they know that if they are assigned to a training condition, it is an “active” condition).

It is planned to disseminate the trial results via publication in peer-reviewed journals, following ICMJE authorship eligibility guidelines, and via conference presentations, and to make materials and data available on publication of the associated study results, via a publicly available data repository, with the exception of data that could compromise participant anonymity and materials subject to copyright or other restrictions, or available elsewhere. If the current study suggests that a fully powered RCT would be feasible and warranted, funding for such a trial could be sought.

## Abbreviations

ANOVA: Analysis of variance
BDI-II: Beck Depression Inventory–Second Edition
CBM: Cognitive bias modification
CBT: Cognitive behavior therapy
CCT: Cognitive Control Training
CEQ: Credibility/Expectancy Questionnaire
DARS: Dimensional Anhedonia Rating Scale
DIPS-/Mini-DIPS-OA: Diagnostisches Interview für psychische Störungen–Open Access
DMC: Data Monitoring Committee
DSM-IV: Diagnostic and Statistical Manual for Mental Disorders, 4th Edition
GAD-7: Generalized Anxiety Disorder Scale
IAT: Implicit Association Test
ISI: Inter-stimulus interval
KW: Katharina Westermann
MLW: Marcella L. Woud
PANAS-P: Positive and Negative Affect Schedule–21-item positive subscale
PANAS-X: Positive and Negative Affect Schedule–Expanded Form
PIT: Prospective Imagery Test
PMH: Positive Mental Health Scale
PMIT: Positive Mental Imagery Training
QIDS-SR: Quick Inventory of Depressive Symptomatology–Self-Report
RCT: Randomized controlled trial
SEB: Simon E. Blackwell
SHAPS: Snaith-Hamilton Pleasure Scale
SST: Scrambled Sentences Test
STIAT: Single-Target Implicit Association Test
TAU: Treatment As Usual
TEPS: Temporal Experience of Pleasure Scale
TN: Torsten Neher
